# Customized Trajectory Optimization and Compliant Tracking Control for Passive Upper Limb Rehabilitation

**DOI:** 10.3390/s23156953

**Published:** 2023-08-04

**Authors:** Liaoyuan Li, Jianhai Han, Xiangpan Li, Bingjing Guo, Xinjie Wang

**Affiliations:** 1 School of Mechatronics Engineering, Henan University of Science and Technology, Luoyang 471023, China; hkdlly@stu.haust.edu.cn (L.L.); xiangpanli@haust.edu.cn (X.L.); bingjing@haust.edu.cn (B.G.); 2 Henan Provincial Key Laboratory of Robotics and Intelligent Systems, Luoyang 471000, China; 3 Collaborative Innovation Center of Machinery Equipment Advanced Manufacturing of Henan Province, Luoyang 471003, China; 4 School of Mechatronics Engineering, Zhengzhou University of Light Industry, Zhengzhou 450001, China; wangxinjie@zzuli.edu.cn

**Keywords:** upper limb rehabilitation, trajectory customization, compliant control, error subdivision

## Abstract

Passive rehabilitation training in the early poststroke period can promote the reshaping of the nervous system. The trajectory should integrate the physicians’ experience and the patient’s characteristics. And the training should have high accuracy on the premise of safety. Therefore, trajectory customization, optimization, and tracking control algorithms are conducted based on a new upper limb rehabilitation robot. First, joint friction and initial load were identified and compensated. The admittance algorithm was used to realize the trajectory customization. Second, the improved butterfly optimization algorithm (BOA) was used to optimize the nonuniform rational B-spline fitting curve (NURBS). Then, a variable gain control strategy is designed, which enables the robot to track the trajectory well with small human–robot interaction (HRI) forces and to comply with a large HRI force to ensure safety. Regarding the return motion, an error subdivision method is designed to slow the return movement. The results showed that the customization force is less than 6 N. The trajectory tracking error is within 12 mm without a large HRI force. The control gain starts to decrease in 0.5 s periods while there is a large HRI force, thereby improving safety. With the decrease in HRI force, the real position can return to the desired trajectory slowly, which makes the patient feel comfortable.

## 1. Introduction

With global aging and the increasing number of patients with limb motor dysfunction caused by nerve injury, how to meet the urgent rehabilitation demand and improve the quality of life of patients is a key issue to be solved [1]. Compared to the traditional manual rehabilitation method by therapists, the robot-assisted rehabilitation method has the advantages of high repeatability, high precision, and accurate quantitative evaluation, which has become a research hotspot worldwide [2]. Clinical results have shown that passive rehabilitation training can promote neural remodeling in the initial post-stroke period [3], which helps the muscles of the affected limb regain the ability to contract spontaneously. Traditional rehabilitation training is effective due to the practical experience of the physiotherapist and the real-time interaction. At present, the training trajectory of most rehabilitation robots can only be of some regular curves represented by clear mathematical functions. On the other hand, the training strategy cannot deal with a large HRI force, which might exert damage to the patient. Therefore, it is needed to study the customization of training trajectory and compliant control of passive rehabilitation training.

Emken et al. [4] proposed a teaching control strategy for a lightweight two-degree-of-freedom (DOF) lower limb rehabilitation robot. Feng et al. [5] fixed an accelerometer on the lower limbs to collect data during the training stage as expected input for rehabilitation training. Morita et al. [6] adopted an impedance control strategy to drag upper limb rehabilitation robots and used the least square method to fit the original noise data to approximate the intention of the rehabilitation physician. You et al. [7] designed a torque control algorithm based on the self-developed DC motor, where the weight of the robot arm and the joint friction torque of the robot are compensated to achieve easy dragging. Yang et al. [8] directly used the admittance algorithm in Cartesian space to calculate the inverse joint position solution to drag the lower limb rehabilitation robot, without optimizing and tracking the personalized trajectory. However, the doctor and the patient’s hands are coupled to the end of the robot, inevitably resulting in an unsmooth trajectory.

As for trajectory optimization, it is generally based on polynomials, including time-optimal, energy-optimal, and acceleration-optimal methods [9,10]. In this paper, the raw trajectory points are first compressed using the Douglas–Puke method [11] and then the NURBS curve [12] is used to interpolate between the compressed points. Dong improved the solution process of the B-spline for the joint trajectory fitting of the 6R robot [13]. Mei optimized the end trajectory of the 6-DOF high-speed parallel robot by combining the joint minimum acceleration and the B-spline curve. They focused on reducing the acceleration of the joint and the fitting trajectory has a large deformation relative to the original trajectory [14]. However, there is no smoothness optimization of the fitting trajectory.

As for the trajectory tracking control, Wu et al. designed a fuzzy sliding mode controller to achieve position tracking of the exoskeleton upper limb rehabilitation robot [15]. The physiotherapist can manually adjust the admittance parameters of the outer loop according to the patient’s condition so that the HRI force is included in the position control. Mushage et al. [16] designed a fuzzy neural network and an error-adaptive nonlinear controller based on state observation to track the trajectory of the 5DOFs upper limb exoskeleton and simulate the performance of the controller. Li et al. [17] designed a robust anti-interference controller to improve the trajectory tracking accuracy of the robot. Jia et al. [18] combined RBF neural network and PID for trajectory tracking control of a lower extremity exoskeleton robot. It can be seen that most researchers aim to improve the tracking accuracy with the complex controller. Good trajectory tracking ability can ensure the training effect, but the larger rigidity may make the patient feel uncomfortable or even injured once there is a large HRI force. Therefore, in a normal situation, the upper limb rehabilitation robot should enable trajectory tracking and, in an emergency, it should ensure safety. Trigili, Emilio et al. [19] use series elastic joint elements to achieve compliance of the rehabilitation robot. Miao et al. [20] design a position controller for passive training with a bilateral end-effector upper limb rehabilitation robot and an adaptive variable parameter controller to achieve compliance. Guo et al. [21] use a reinforcement learning algorithm to design a variable admittance control algorithm to achieve rehabilitation training matching the stiffness characteristics of patients’ lower limbs. Among them, the structure and modeling of special flexible components are complex and will have errors. Other compliance control algorithms can modify the parameters online but the algorithms are slightly complex for passive rehabilitation training. Furthermore, the return movement after compliance was not considered before.

Therefore, based on the self-developed 3DOFs end-effector upper limb rehabilitation robot, the methods of trajectory demonstration and optimization are proposed here. A variable gain control strategy is designed, enabling the robot to track the trajectory well with small HRI forces and comply with large HRI forces to ensure safety. Moreover, when the HRI force is reduced with a large position error, the position can also return to the desired trajectory with subdivision error, which means that the return action is not too rushed. It will be comfortable for the patient to continue the unfinished passive training.

## 2. Materials and Methods

### 2.1. Upper Limb Rehabilitation Robot System

**Figure 1:**
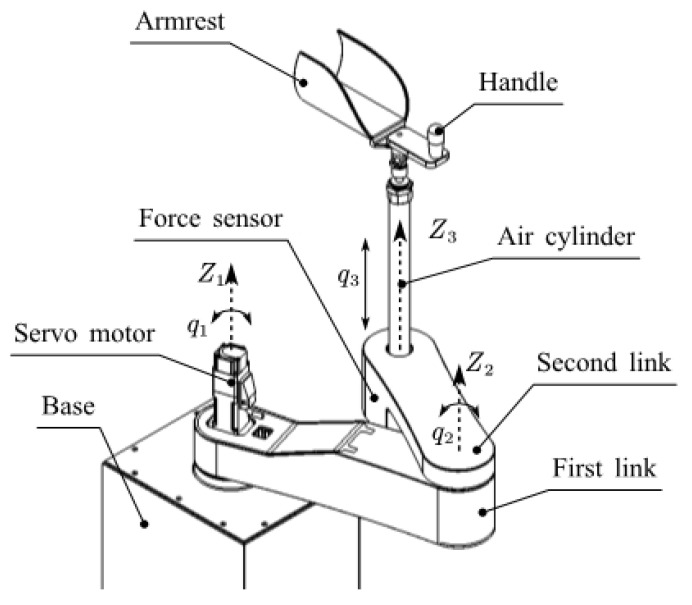
Robot structure.

**Figure 2:**
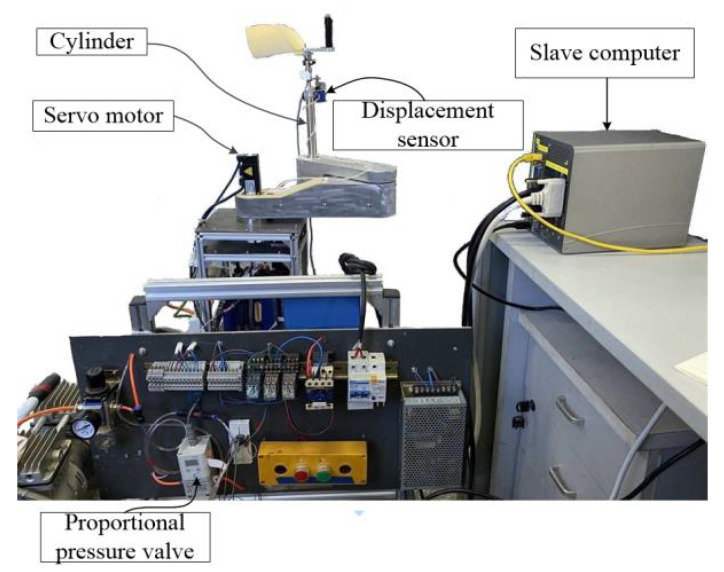
The apparatus of the rehabilitation robot system.

**Figure 3:**
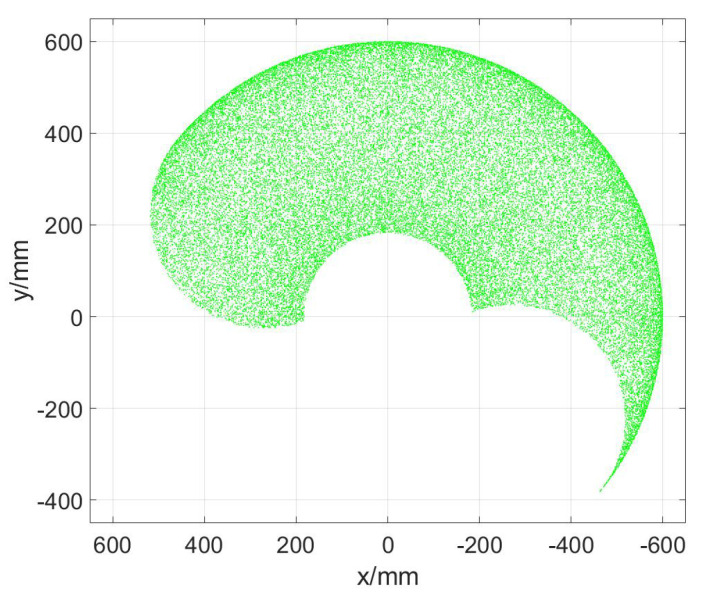
The horizontal workspace of the robot.

The upper limb rehabilitation robot used in this paper is a self-developed 3DOF end-effector upper limb rehabilitation robot, which is mainly used for the training of the shoulder and elbow joints. It includes two horizontal rotating joints and a vertical prismatic joint. Figure 1 is a brief diagram of its structure, where $Z_{1}$, $Z_{2}$, and $Z_{3}$ represent the axes of the three joints, and $q_{1}$, $q_{2}$, and $q_{3}$ are the position variables of the three joints. The first two rotating joints are driven by two AC servomotors equipped with absolute encoders, and the third joint is driven by a double-acting cylinder. A displacement sensor is installed at the end of the cylinder and a three-dimensional force sensor is installed under the cylinder. Figure 2 is the prototype of the rehabilitation robot system. The program is developed with MATLAB software on the host computer. The slave computer executes the compiled control algorithm. The cylinder output force is controlled by a proportional pressure valve. Because of the overall height of the robot, the total stroke of the cylinder is 150 mm, which limits the training range of the shoulder joint in the sagittal plane. Furthermore, to ensure safety, the two rotating joints are mechanically limited. The working range of the robot in the vertical direction is the stroke of the cylinder which is very clear. Therefore, only the working space in the horizontal plane is shown in Figure 3.

### 2.2. Trajectory Customization

While customizing, the patient and the therapist simultaneously exert an interactive force at the end; the resultant force is as follows:

(1) $$\begin{array}{c} F_{\mathrm{int}}=F_{t}+F_{p} \end{array}$$

where $F_{t}$ represents the force exerted by the physiotherapist and $F_{p}$ represents the force of the patient.

Therefore, part of $F_{t}$ should counteract $F_{p}$ so that the robot can move as the therapist wishes. The force $F_{p}$ is variable and difficult to predict. However, for patients who have almost completely lost the ability to move, their interaction force can be assumed to be 0. The robot will move to utilize a force-based admittance control algorithm.

To realize effortless teaching, the rotational friction $F_{f}$ must be compensated. The interaction force $F_{\mathrm{int}}$ is then converted into torques $\tau_{h}$ of joints according to Equation (2), where J is the Jacobin matrix of the robot. Then the admittance algorithm (3) is applied to generate the desired input of the joint position. Therefore, the robot can rotate when there are interaction forces.

(2) $$\begin{array}{c} \tau_{h}=J^{T}F_{\mathrm{int}} \end{array}$$

(3) $$\begin{array}{c} k\Delta q=\tau_{h} \end{array}$$

where k is the admittance coefficient and Δq is the increment of the joint angle.

Regarding the prismatic joint, the static friction force will be tested and compensated according to Equation (4). In addition, the vertical load at the end can be detected and compensated with the help of the force sensor. The force applied by the physiotherapist $F_{\mathrm{PZ}}$ is detected by the force sensor at the end and then the amount of pressure change in the rodless chamber is calculated by Equation (5). Therefore, the cylinder with different loads can be easily moved.

(4) $$\begin{array}{c} F_{f}=15\mathrm{sgn}\left(F_{\mathrm{PZ}}\right) \end{array}$$

where $F_{\mathrm{PZ}}$ represents the vertical force exerted by the therapist.

(5) $$\begin{array}{c} F_{\mathrm{PZ}}=\Delta P\times A_{1} \end{array}$$

where $A_{1}$ is the area of the cylinder’s piston, ΔP indicates the amount of change in pressure.

### 2.3. Trajectory Interpolation

A *k*th-degree NURBS curve defined by *n* + 1 polygon control vertices can be represented as a segmented rational polynomial function. The point on the NURBS curve for a given parameter *u* is obtained as follows.

(6) $$P\left(u\right)=\frac{\sum_{i=0}^{n}\omega_{i}d_{i}N_{i,k}\left(u\right)}{\sum_{i=0}^{n}\omega_{i}N_{i,k}\left(u\right)}$$

where $\omega_{i}$ is the weight factor, which is related to the control points $d_{i}$. The larger the value of the weight factor, the closer the curve is to the control vertex. The first and last weight factors $\omega_{0},\omega_{n}>0$ and the rest $\omega_{i}$ ≥ 0, which prevent the denominator from being zero, retain the convex wrapping nature and do not degrade the curve to a point due to the weight factor. $N_{i,k}\left(u\right)$ is a *k*th-degree normal B-spline basis function defined by a non-periodic and nonuniform node vector $U=\left[u_{0},u_{1},\cdots ,u_{n+k+1}\right]$ deduced from the Cox–De Boor recursive formula expressed as follows.

(7) $$\left\{\begin{array}{l} N_{i,0}=\left\{\begin{array}{ll} 1 & u_{i}⩽u⩽u_{i+1}\\ 0 & \;\mathrm{Otherwise}\; \end{array}\right.\\ N_{i,k}\left(u\right)=\frac{u-u_{i}}{u_{i+k}-u_{i}}N_{i,k-1}\left(u\right)+\frac{u_{i+k+1}-u}{u_{i+k+1}-u_{i+1}}N_{i+1,k-1}\left(u\right)\\ \;\mathrm{Define}\;\frac{0}{0}=0 \end{array}\right.$$

To make a *k*th-degree NURBS curve pass through a given set of points $P_{i}\left(i=0,1,\ldots ,n\right)$, it is necessary to ensure that the first and last points of the curve coincide with the points $P_{0}$ and $P_{n}$, while ensuring that the nodes $u_{i+k}\left(i=0,1,\ldots ,n\right)$ in the curve definition field correspond to $P_{i}$ one-to-one. A *k*th-degree NURBS curve with *n* segments will be defined by n+3 control points $D_{i}\left(i=0,1,\ldots ,n+2\right)$, the weight factors $\omega_{i}$, and the node vector $U=\left[u_{0},u_{1},\ldots ,u_{n+k+3}\right]$.

To parameterize compressed points $P_{i}$, three parameterization methods, named uniform parameterization, cumulative chord length parameterization, and centripetal parameterization, can be used. The second method can accurately reflect the distribution of the points $P_{i}$ and the fitting accuracy is high [22]. Therefore, the cumulative chord length parameterization is used to parameterize the points $P_{i}$. The method is represented below.

(8) $$\left\{\begin{array}{l} u_{0}=u_{1}=u_{2}=u_{3}=0\\ u_{i+3}=u_{i+2}+\left|P_{i}-P_{i-1}\right|/\sum_{i=1}^{n}\left|P_{i}-P_{i-1}\right|\\ u_{n+3}=u_{n+4}=u_{n+5}=u_{n+6}=1 \end{array}\right.$$

Variables that affect the fitting effect of the NURBS curve are control points, curve nodes, and weight factors [23]. In the experiment, to simplify the calculation, the weight factors are set to 1. The different compression thresholds lead to different initial via points as well as the shape and smoothness of the fitted NURBS curves. Therefore, an intelligent optimization algorithm, with curvature as the objective function and compression threshold as the variable for optimization, is proposed to produce a continuous smooth curve as the training trajectory. The curvature of a point on the NURBS curve is written as follows.

(9) $$\kappa_{c}=\frac{∥\dot{P}\left(u\right)\times \ddot{P}\left(u\right)∥}{{∥\dot{P}\left(u\right)∥}^{3}}$$

where $\dot{P}\left(u\right)$ and $\ddot{P}\left(u\right)$ are the first and second derivatives of the curve that can be calculated according to Leibniz’s rule. Simplify Equation (6) as follows.

(10) $$P\left(u\right)=\frac{A(u)}{W(u)}$$

Therefore, the *k*th-order derivative is deduced as

(11) $$P^{\left(k\right)}\left(u\right)=\frac{A^{\left(k\right)}\left(u\right)-\sum_{i=1}^{k}C_{k}^{i}W^{\left(i\right)}\left(u\right)P^{\left(k-i\right)}\left(u\right)}{W(u)}$$

Finally, the objective function is written as

(12) $$obj=\sum_{j=0}^{m}\kappa_{c}\left(j\right)$$

where *m* represents the number of interpolation points on the curve.

In addition, the trajectory should be limited to the workspace of the robot. According to the inverse kinematics model of the robot, the position q of three joints can be obtained from the coordinate points in Cartesian space. The optimization constraint is expressed as:

(13) $${\underline{q}}_{i}\leq q_{i}\leq {\bar{q}}_{i}\;i=1,2,3$$

where ${\underline{q}}_{i}$ and ${\bar{q}}_{i}$ represent the minimum and maximum limits of the joint *i*.

### 2.4. Optimization Algorithm

The Butterfly Optimization Algorithm (BOA) is a new type of metaheuristic group intelligence optimization algorithm inspired by the foraging and courtship behavior of butterflies in nature based on sensed fragrance [24]. It includes global search and local search. Compared with some existing metaheuristic algorithms, the basic BOA operation is simple, with few adjusted parameters and good robustness, and it has achieved good results in the preliminary application of engineering practice [25].

The fragrance is formulated as a function of the physical intensity of the stimulus as follows:

(14) $$f=cI^{a}\;ac\in \left[0,1\right]$$

where *f* is the perceived magnitude of the fragrance, that is, how strong the fragrance is perceived by other butterflies, *c* is the sensory modality, *I* is the stimulus intensity, and *a* is the power exponent dependent on the modality, which accounts for the variable degree of absorption. Generally, *c* = 0.01 and *a* = 0.1. In the case of a maximization problem, the intensity can be proportional to the objective function.

Before the BOA enters the local or global search, the algorithm randomly generates the locations of individuals and produces their respective scents accordingly. Each butterfly moves to the current global optimal position $g^{*}$ during the global search phase. The global searching rule is written as:

(15) $$x_{i}^{t+1}=x_{i}^{t}+f_{i}\left(r_{1}^{2}g^{*}-x_{i}^{t}\right)$$

where $x_{i}^{t}$ is the position of the *i*th butterfly in the *t*th iteration. Here, $g^{*}$ represents the current best position. The fragrance of the *i*th butterfly is represented by $f_{i}$ and $r_{1}$ is a random number in [0, 1].

The local search phase can be represented as follows.

(16) $$x_{i}^{t+1}=x_{i}^{t}+f_{i}\left(r_{1}^{2}x_{j}^{t}-x_{k}^{t}\right)$$

where $x_{j}^{t}$ and $x_{k}^{t}$ are positions of the *j*th and *k*th butterflies, respectively. In the butterfly foraging, whether it is in the local search phase or the global search phase, it is determined by the switching probability $P_{\mathrm{static}}$ = 0.8. Each iteration compares a random number $r_{2}$∈ [0, 1] with $P_{\mathrm{static}}$. The final position update formula of the butterfly algorithm is as follows.

(17) $$x_{i}^{t+1}=\left\{\begin{array}{ll} x_{i}^{t}+f_{i}\left(r_{1}^{2}g^{*}-x_{i}^{t}\right)\; & r_{2}\leq P_{\mathrm{static}}\\ x_{i}^{t}+f_{i}\left(r_{1}^{2}x_{j}^{t}-x_{k}^{t}\right)\; & r_{2}>P_{\mathrm{static}} \end{array}\right.$$

To solve the problems of slow convergence speed, low convergence accuracy, and easily falling into local optima of standard BOA, many researchers have improved the algorithms [26,27,28]. The improvements deal with multidimensional optimization problems. In this study, the trajectory data compression algorithm and BOA are combined to reduce the optimization problem from three-dimensional to one-dimensional. Therefore, in the iterative operation process, dynamic switching probability and t-variation strategies are used to improve the convergence speed and accuracy of BOA.

The idea of dynamic switching probability can be expressed in the following expression.

(18) $$P_{d}={\left(\frac{T_{\max}-\hat{n}}{T_{\max}}\right)}^{3}$$

where $T_{\max}$ represents the maximum number of iterations and $\hat{n}$ represents the current number of iterations. In the iterative process, random numbers $r_{1}^{2}$ are replaced with a t-distribution function, preventing local optimization and improving the convergence speed.

The probability density function of the standard t-distribution is as follows.

(19) $$\begin{array}{cc} f(t) & =\frac{\Gamma (\frac{n+1}{2})}{\sqrt{n\pi }\Gamma \left(\frac{n}{2}\right)}{\left(1+\frac{t^{2}}{n}\right)}^{-\frac{n+1}{2}}\\ \; \end{array}$$

where *n* is the freedom of the gamma function Γ.

With the number of iterations of BOA correlated, Equation (19) can be rewritten as

(20) $$f\left(\bar{t}\right)=\frac{\Gamma (\frac{n+1}{2})}{\sqrt{n\pi }\Gamma \left(\frac{n}{2}\right)}{\left(1+\frac{{\left(\hat{n}/T_{max}\right)}^{2}}{n}\right)}^{\frac{n+1}{2}}$$

where $\bar{t}=\hat{n}/T_{max}$. In the experiment, let *n* = 20, the improved BOA algorithm is written as follows.

(21) $$x_{i}^{t+1}=\left\{\begin{array}{ll} x_{i}^{t}+f_{i}\left(f\left(\bar{t}\right)g^{*}-x_{i}^{t}\right),\; & r_{2}\leq P_{d}\\ x_{i}^{t}+f_{i}\left(f\left(\bar{t}\right)x_{j}^{t}-x_{k}^{t}\right),\; & r_{2}>P_{d} \end{array}\right.$$

**Figure 4:**
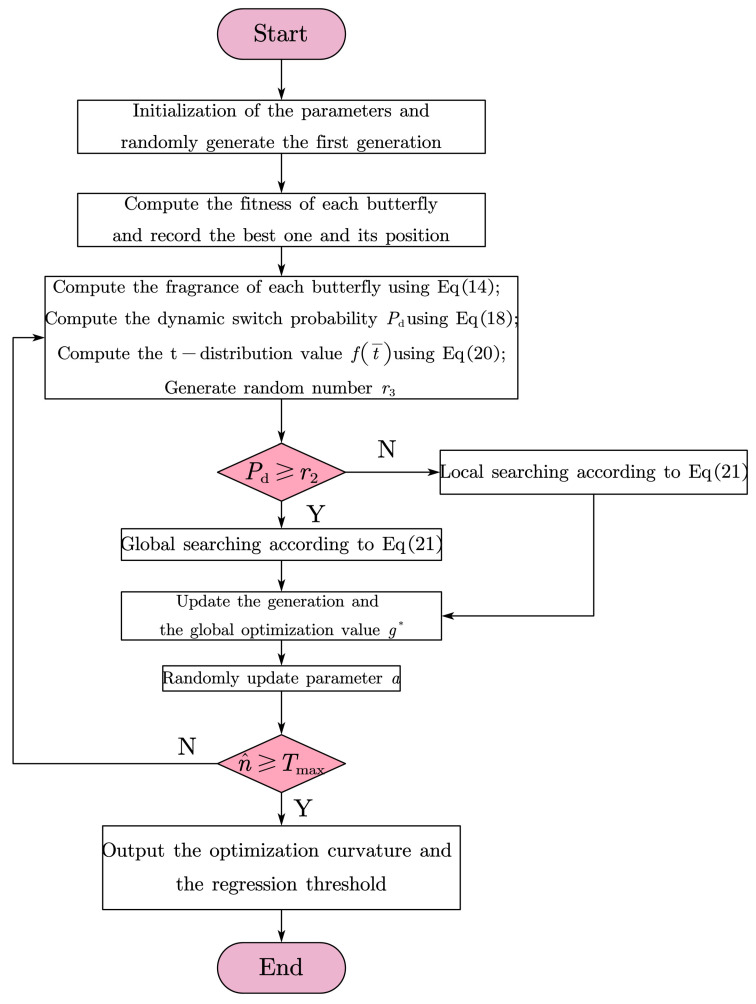
The optimization process of the improved BOA.

**Figure 5:**
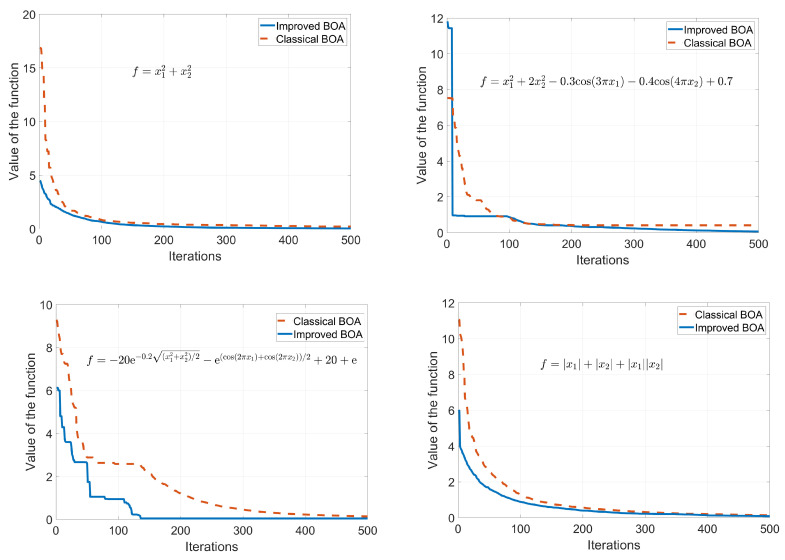
Optimization of the test functions.

**Figure 6:**
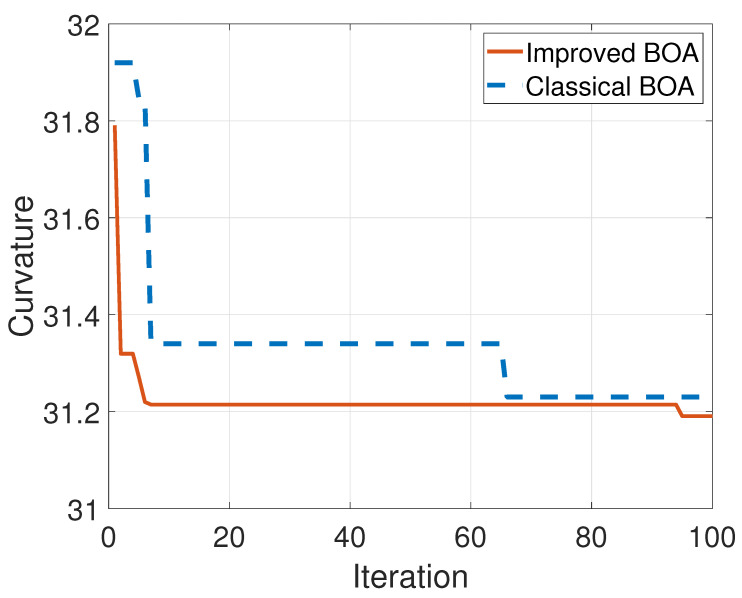
The curvature optimization process.

The flow chart of the optimization is shown in Figure 4. Chose four test functions to test the performance of the proposed algorithm. The functions are listed in Table 1. The optimization results are shown in Figure 5. The iterative optimization processes of the improved BOA and the classical BOA are shown in Figure 6. The minimum sum of curvature of the improved BOA is 31.1908 after 100 iterations and the corresponding optimal compression threshold is 24.9157. The results of the classical BOA are 31.3277 and 24.042. It can be seen that the improved algorithm has a better regression speed and accuracy. The blue solid line represents the proposed improved algorithm. The red dashed line is the classical algorithm. It can be seen that improved BOA is better than the classical BOA.

**Figure 7:**
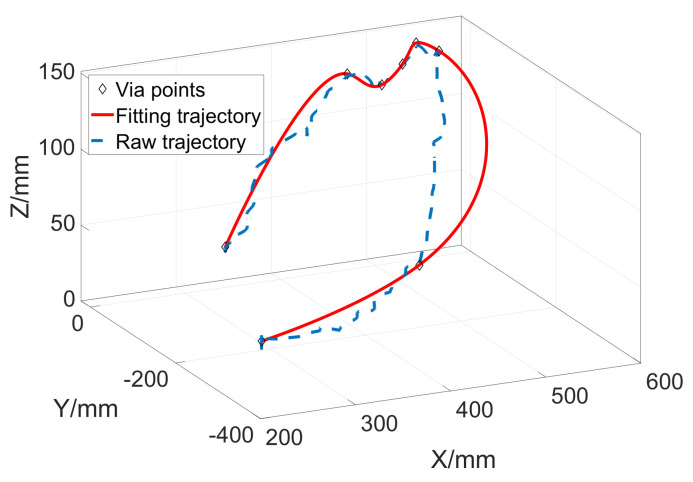
The NURBS interpolation curves of an arbitrary spatial trajectory.

The interpolation curve is shown in Figure 7, where the blue dotted line represents the raw trajectory and the red line represents the optimized interpolation curve.

### 2.5. Trajectory Tracking Controller

#### 2.5.1. RBF Net-Based Controller

To realize the training motion planned by the rehabilitation physician, passive rehabilitation training requires good trajectory tracking performance under the premise of ensuring safety. First, a sliding mode controller based on the RBF approximation is designed to ensure good tracking performance in the training process. The dynamic equation of the *n*-joint robot is as follows.

(22) $$M\left(q\right)\ddot{q}+C\left(q,\dot{q}\right)\dot{q}+G\left(q\right)+F\left(\dot{q}\right)+\tau_{d}=\tau$$

where M(q) is the n×n positive-definite inertia matrix, $C(q,\dot{q})$ is the n×n Coriolis matrix, G(q) is an n×1 vector of gravity forces, $F(\dot{q})$ is an n×1 vector of friction forces, $\tau_{d}$ is the unknown applied interference and satisfies $∥\tau_{d}∥\leq d$, d is the upper bound of $\tau_{d}$, and τ is the control input. Define the tracking error as follows.

(23) $$e=q_{d}-q$$

Define the sliding surface as Equation (24)

(24) $$r=\dot{e}+\Lambda e$$

where $\Lambda =\Lambda^{T}>0$. Substitute (23) into (24); we have the following expression.

(25) $$\dot{q}=-r+{\dot{q}}_{d}+\Lambda e$$

Then, by substituting (25) and its derivative into (22), we obtain the simplified expression

(26) $$M\left(q\right)\dot{r}=-C\left(q,\dot{q}\right)r-\tau +Q\left(x\right)+\tau_{d}$$

where $Q\left(x\right)=M\left(q\right)\left({\ddot{q}}_{d}+\Lambda \dot{e}\right)+C\left(q,\dot{q}\right)\left({\dot{q}}_{d}+\Lambda e\right)+G\left(q\right)+F\left(\dot{q}\right)$. This is the model uncertainty, which needs to be approximated. Since the RBF network has universal approximation characteristics, the RBF neural network is used to approximate the unknown nonlinear function Q(x) and the RBF network algorithm is defined as follows.

(27) $$\left\{\begin{array}{l} \varphi_{j}=\exp\left(\frac{{∥x-c_{j}∥}^{2}}{2\sigma_{j}^{2}}\right)\\ Q\left(x\right)=W^{*\;T}\varphi \left(x\right)+\varepsilon \end{array}\right.$$

where x is the input of the network, *j* is the *j*th node of the implicit layer of the network, φ(x) is the output of the Gaussian function of the network, and $W^{*}$ is the ideal weight of the network. The approximation error of the network is ε and $∥\varepsilon ∥\leq \varepsilon_{N}$.

The input vector of the network is $x=\left[\begin{array}{lllll} e^{T} & {\dot{e}}^{T} & q_{d}^{T} & {\dot{q}}_{d}^{T} & {\ddot{q}}_{d}^{T} \end{array}\right]$. The robot control input is designed as below.

(28) $$\tau =\hat{Q}\left(x\right)+K_{v}r-v$$

where $K_{v}$ is a diagonal matrix with each element larger than 0 and $\hat{Q}\left(x\right)={\hat{W}}^{T}\varphi \left(x\right)$ is the estimation output of the network. Define $\tilde{W}=W^{*}-\hat{W}$ and then we have $Q\left(x\right)-\hat{Q}\left(x\right)={\tilde{W}}^{T}\varphi \left(x\right)+\varepsilon$. Substituting (28) into (26), we obtain

(29) $$M\left(q\right)\dot{r}=-\left(K_{v}+C\left(q,\dot{q}\right)\right)r+\zeta_{1}$$

where $\zeta_{1}={\tilde{W}}^{T}\varphi \left(x\right)+\left(\varepsilon +\tau_{d}\right)+v$.

Define the Lyapunov function as below.

(30) $$V=\frac{1}{2}r^{T}M\left(q\right)r+\frac{1}{2}\mathrm{tr}\left({\tilde{W}}^{T}\Xi^{-1}\tilde{W}\right)$$

where $\mathrm{tr}\left({\tilde{W}}^{T}\Xi^{-1}\tilde{W}\right)$ is the trace of the matrix ${\tilde{W}}^{T}\Xi^{-1}\tilde{W}$ and Ξ is a positive diagonal matrix. The derivative of *V* is written as

(31) $$\dot{V}=r^{T}M\left(q\right)\dot{r}+\frac{1}{2}r^{T}\dot{M}\left(q\right)r+\mathrm{tr}\left({\tilde{W}}^{T}\Xi^{-1}\dot{\tilde{W}}\right)$$

Substitute (29) and $\dot{M}\left(q\right)-2C\left(q,\dot{q}\right)=0$ into (31) to obtain the following expression.

$$\dot{V}=-r^{T}K_{v}r+\mathrm{tr}{\tilde{W}}^{T}\left(\Xi^{-1}\dot{\tilde{W}}+\varphi r^{T}\right)+r^{T}\left(\varepsilon +\tau_{d}+v\right)$$

To make the system stable, design the adaptation law as $\dot{\tilde{W}}=-\dot{\hat{W}}=-\Xi \varphi \left(x\right)r^{T}$ with Ξ>0. Additionally, design a robust term $v=-\left(\varepsilon_{N}+d\right)\mathrm{sgn}\left(r\right)$ so that we can obtain $\dot{V}=-r^{T}K_{v}r\leq 0$. According to the LaSalle theorem, the closed-loop system is asymptotic and stable.

#### 2.5.2. Variable Gain Strategy

Because the third joint of the robot is perpendicular to the first two rotating joints, the motion is decoupled between them, which can be controlled separately. Considering the compressibility of the gas, the position control of the cylinder is controlled by the PID combined with a velocity feedforward controller. Because the third joint is driven by air pressure resulting in compliant properties, only the first two joints are designed with a variable gain strategy to cope with excessive HRI force and ensure the safety of the training.

Now, define the driving torque of the servomotor as follows.

(32) $$\tau_{c}=\tau +\tau_{h}$$

where $\tau_{h}$ is calculated according to Equation (2).

Based on the control strategy mentioned above, the control gain is $K_{v}$ of Equation (28). According to the HRI force $F_{h}$, the variable gain strategy is designed as follows.

(33) $$K_{v}=\gamma e^{-F_{h}^{2}/\sigma_{1}}$$

where γ is a 2 × 2 positive definite diagonal matrix. This determines the maximum of $K_{v}$. $F_{h}$ is the resultant force of $F_{X}$ and $F_{Y}$. This is a scaler. $\sigma_{1}$ is a scaler and determines the working range of $F_{h}$. The smaller the value of $\sigma_{1}$, the smaller $F_{h}$ resulting in a maximum of $K_{v}$.

According to (32) and (33), the value of $K_{v}$ will be small when there is a large HRI force, meaning that $\tau_{h}$ becomes the main driving torque and the robot will move away from the desired trajectory. Once the HRI force decreases, $K_{v}$ increases, returning the real position to the desired trajectory to continue training. But if the value of the control gain is large, the return motion will be very fast, causing an uncomfortable feeling. Therefore, design the position error subdivision strategy so that the trajectory tracking process is gradually completed through small errors.

**Figure 8:**
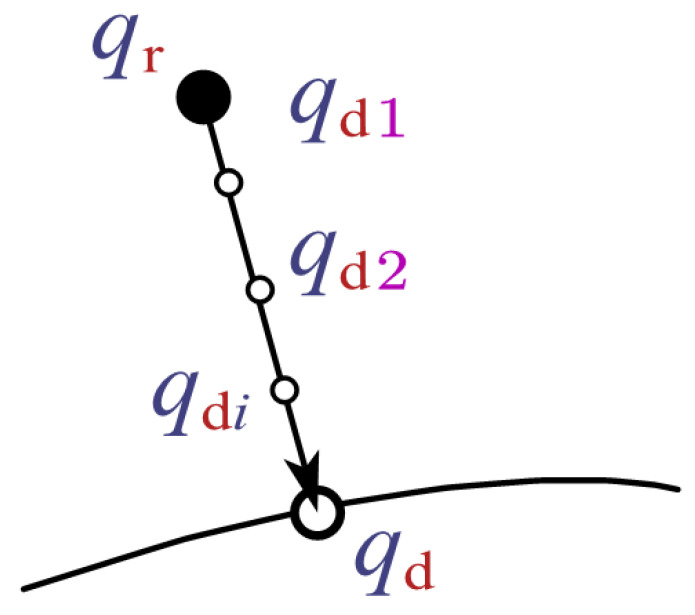
Subdivision of the error.

Assume the expected position of the deviated joint is $q_{d}$, the actual position is $q_{r}$, and the position error is *E*. Set a constant χ to divide the error *E* and each small segment of error will form a transitional expected position $q_{di}$, as shown in Figure 8. The formula is as follows.

(34) $$\left\{\begin{array}{l} q_{di}=q_{r}+E/\chi \\ \chi =\mathrm{ceil}(\lambda |E|) \end{array}\right.$$

where the ceil() function returns the smallest integer greater than or equal to the specified expression. This function makes the parameter χ vary with the error *E*. For instance, in the beginning, *E* is large, making χ large, which ensures the subdivided error is small and the motion is slow. If χ is a fixed constant, the subdivided error will be large in the beginning and very small in the end, which leads to fast motion in the beginning and not being able to return to the desired trajectory in the end. The absolute value of *E* is taken to prevent χ from being zero. As the error in the tracking process always exists, so the minimum of χ is 1. The coefficient λ amplifies |E| to a value that is greater than 1, thereby avoiding the situation where χ is always 1.

**Figure 9:**
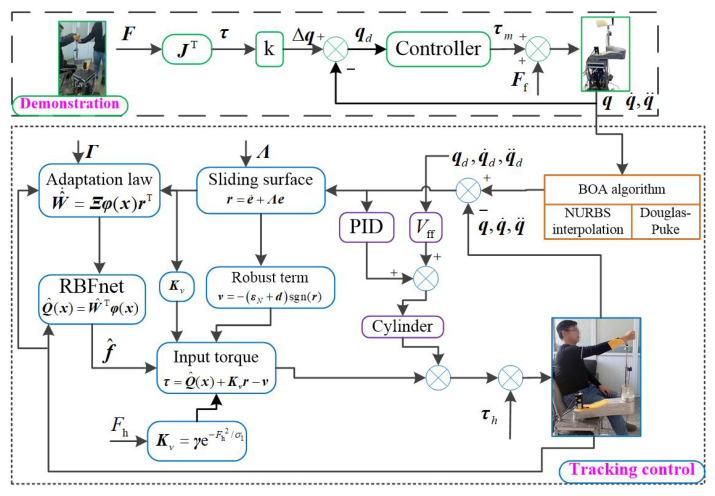
The control algorithm.

The control algorithm proposed here is shown in Figure 9.

## 3. Tracking Experiments and Results

### 3.1. Trajectory Customization Experiment

**Figure 10:**
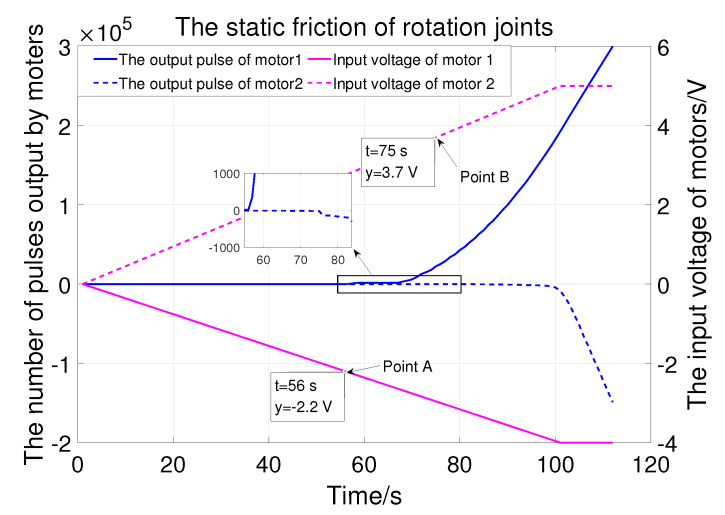
The static friction of joint 1 and joint 2.

The first two joints are driven by servomotors. Different input voltages lead to different output torque. Therefore, ramp signals are used to test the static friction of the rotation joints. As shown in Figure 10, the voltage that drives joint 1 to start rotating is 2.2v (point A) and that for joint 2 is 3.7v (point B). They are used as compensation for the static friction of the rotating joint.

**Figure 11:**
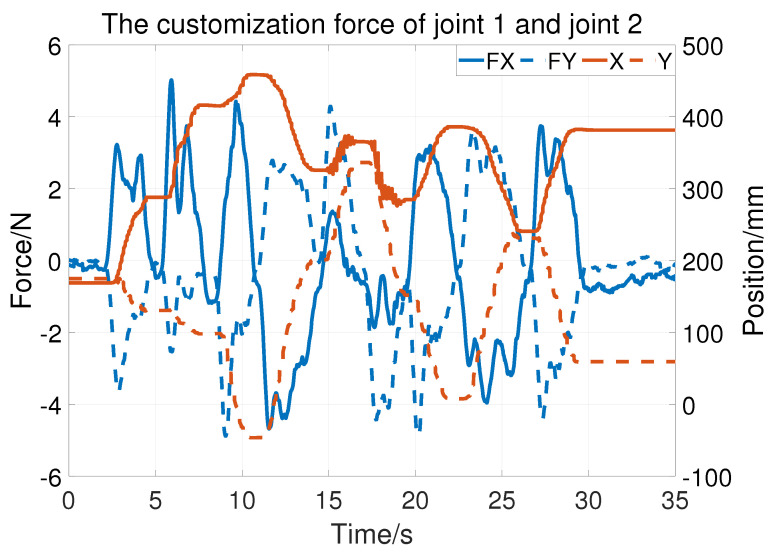
The customization test of joint 1 and joint 2.

Figure 11 shows the customization force of the first two joints. The blue lines represent the force of the *X* and *Y* directions (denoted $F_{X}$ and $F_{Y}$), which are measured by the force sensor. The orange lines represent the position of *X* and *Y*. Some force is still needed to rotate the robot, as the static friction and inertia forces may vary with the robot configurations. However, the largest force is 5.021N, which means that it is easy to rotate the first two joints.

**Figure 12:**
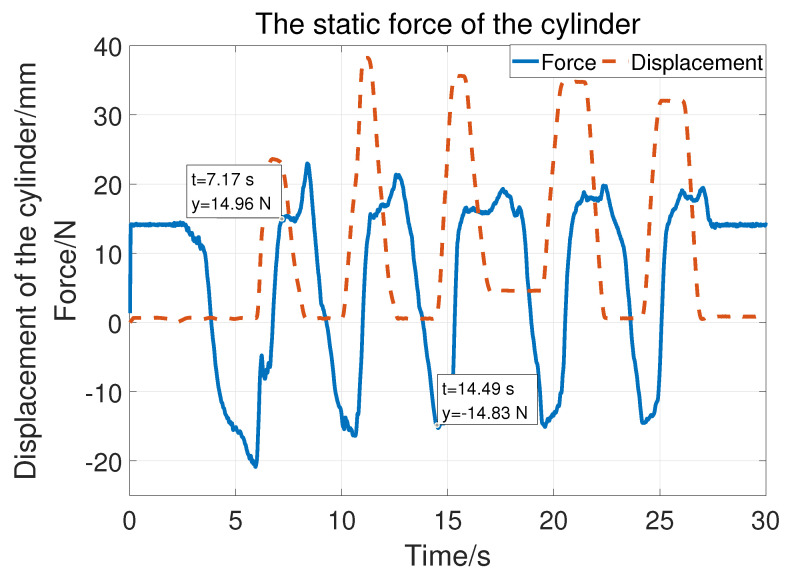
The static friction of joint 3.

**Figure 13:**
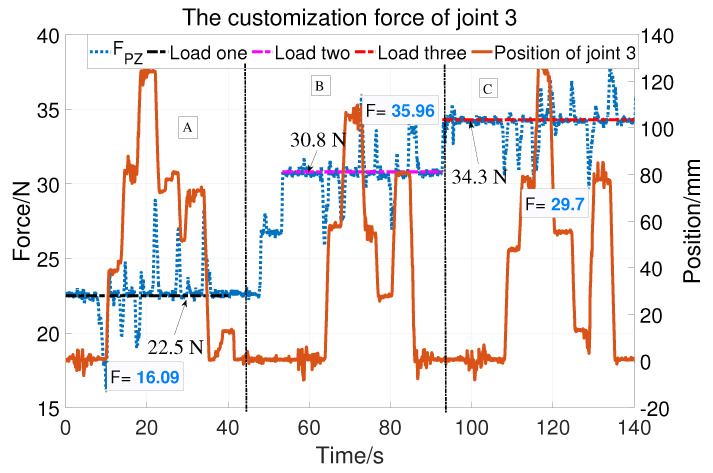
The customization force of joint 3.

Regarding the prismatic joint, the vertical load at the end can be detected and compensated with the help of the force sensor. The static friction force test is shown in Figure 12. The solid blue line is the force and the crest and trough corresponding to the moments when the cylinder starts to move down and up. So, the force can be regarded as the friction force. The static friction is compensated according to Equation (4). Figure 13 is divided into three parts (A, B, and C) showing compensation with three different vertical loads (22.5 N, 30.8 N, and 34.3 N). The orange solid line represents the displacement of the cylinder and the blue line represents the vertical load as well as the customization force. The cylinder can easily stay at different positions with compensation. The maximum customization force is 6.41N of Part A, 5.16 N of Part B, and 4.6N of Part C. That is, the cylinder can be easily moved with different loads.

### 3.2. Trajectory Tracking Experiment

**Figure 14:**
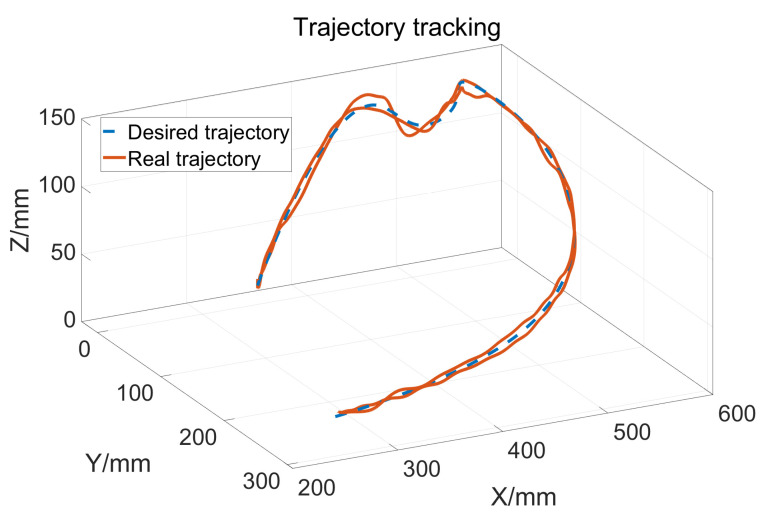
Trajectory tracking results.

**Figure 15:**
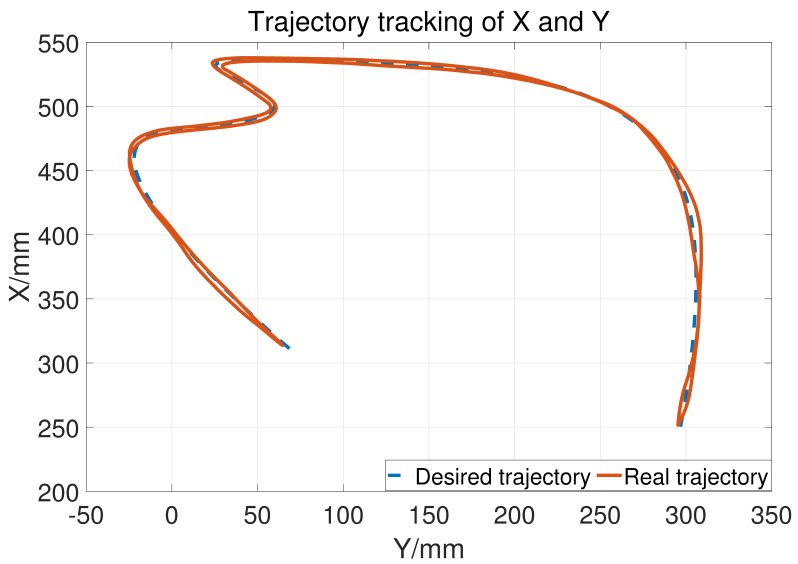
The tracking results in the horizontal plane.

**Figure 16:**
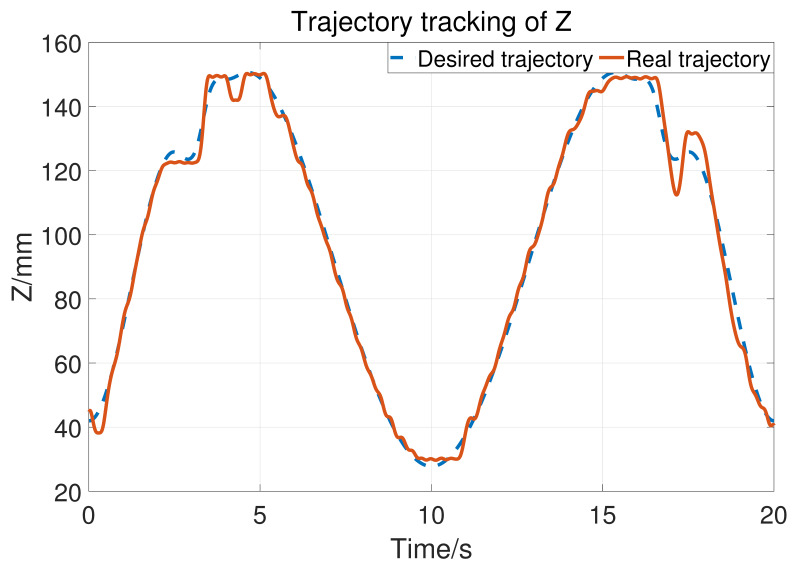
The position tracking of joint 3.

**Figure 17:**
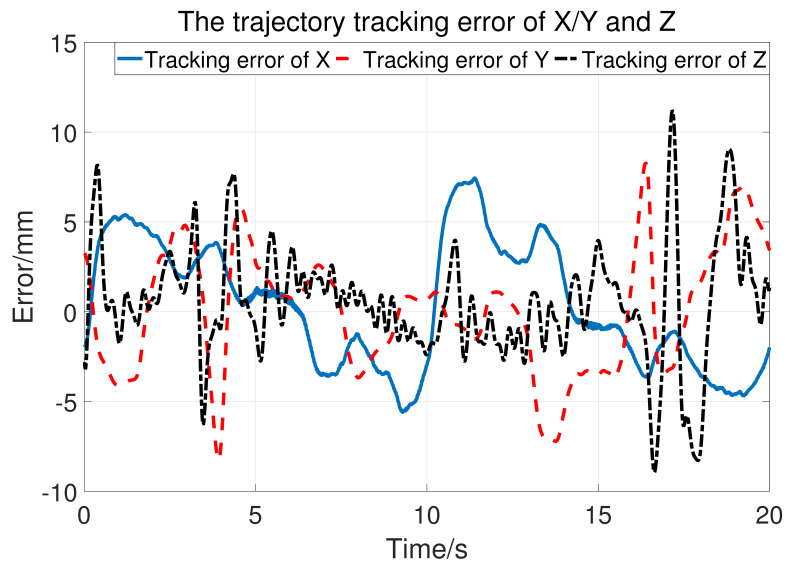
The tracking error in Cartesian space.

Let $a=15,\;b=10,\sigma_{1}=500$, so the upper bound of $K_{v}$ is 25. The two joints use the same control parameter. During the experiment, the subject does not exert an active force and the tracking result is shown in Figure 14. The blue dashed line is the desired trajectory and the orange solid line is the real trajectory. Figure 15 shows the tracking performance in the horizontal plane and Figure 16 displays the cylinder tracking result. It can be seen that the tracking error (Figure 17) is mainly from the third joint. The maximum tracking error is shown in Table 2. From the experiment results, it can be concluded that, without a large HRI force, better trajectory tracking can be achieved, which can meet the needs of passive training.

### 3.3. Compliant Control Experiment

**Figure 18:**
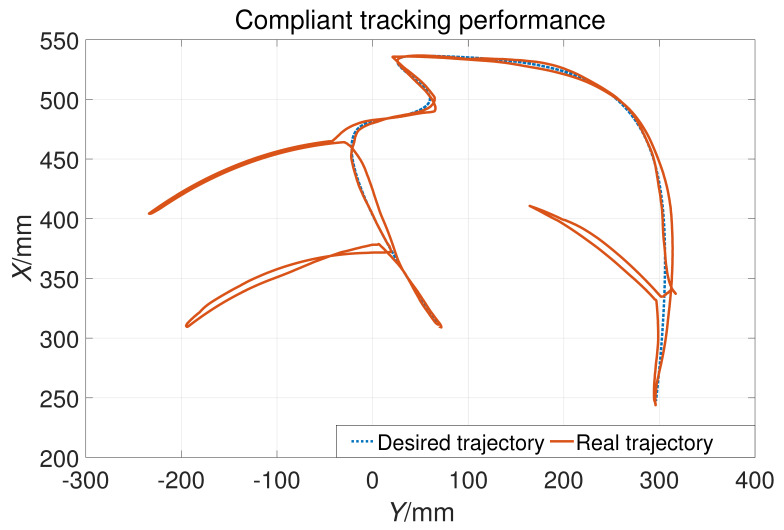
The performance of compliant properties in the horizontal plane.

**Figure 19:**
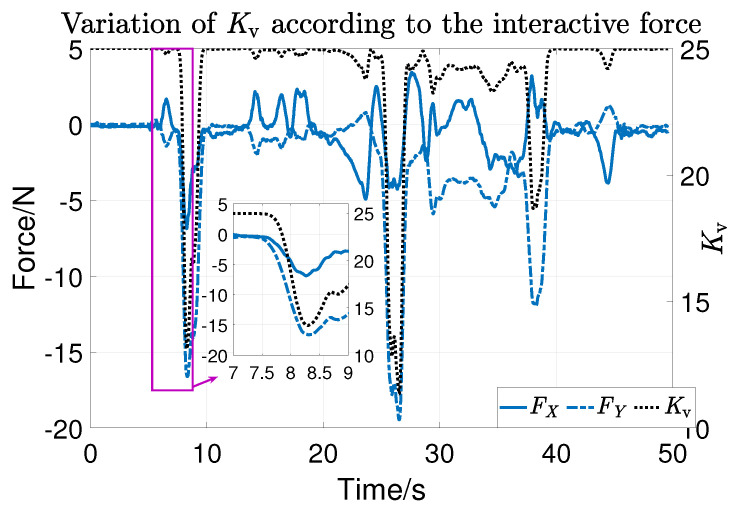
The change in $K_{v}$ according to HRI force.

During the experiment, the subjects randomly applied active force and the tracking results are shown in Figure 18. It can be seen that the actual position (solid orange line) deviates from the expected trajectory (blue dashed line). That is, trajectory tracking can adapt to the interaction forces randomly applied by subjects during the training process, verifying the effectiveness of variable parameters. Figure 19 shows the changes in the control parameter $K_{v}$ caused by the interaction force. The black dotted line represents the change in control parameters, the blue solid line represents the force $F_{X}$ in the *X* direction, and the blue dotted line represents the force $F_{Y}$ in the *Y* direction. Obviously, as the interaction force increases, the control gain decreases from the maximum value of 25. It can be seen from the enlarged figure of first compliance that the response time of impedance parameters to the interaction force is within 0.5 s. At this point, the main torque to drive the robot to move is $\tau_{h}$, so the tracking can be compliant with the interaction force. It also can be seen that $K_{v}$ changes almost simultaneously with the interaction force.

**Figure 20:**
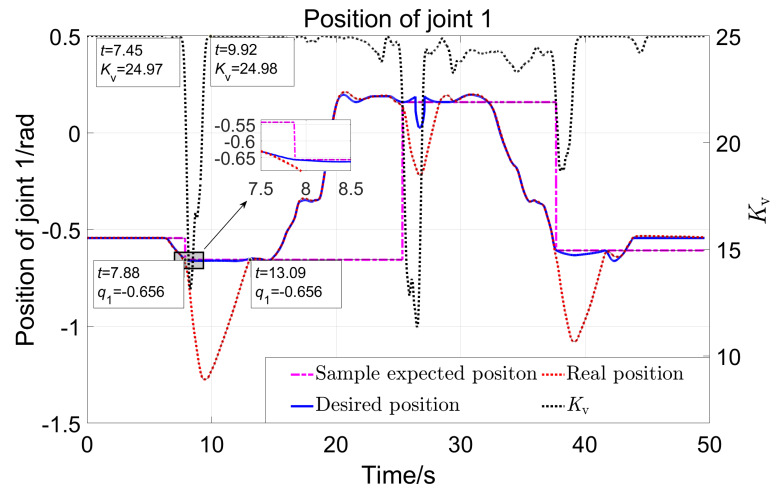
The position of joint 1 with the variation of $K_{v}$.

**Figure 21:**
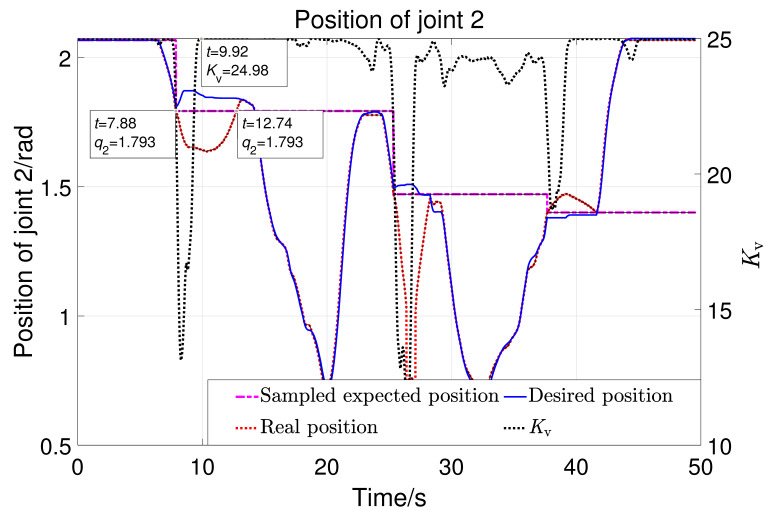
The position of joint 2 with the variation of $K_{v}$.

Figure 20 shows the displacement of robot joint 1 with the variation of the parameter $K_{v}$. The black dotted line in the figure represents the parameter $K_{v}$. The pink stepped dash-dotted line represents the sampled expected position when the position of joint 1 deviates. The solid blue line represents the expected position of joint 1 in real-time. The red dotted line represents the actual position. There were three significant position deviations in the experiment, with three sampled expected positions (−0.656,0.159, −0.608rad). Taking the first large displacement deviation as an example: when t=7.45s, the value of $K_{v}$ begins to decrease. From the enlarged image indicated by the arrow, it can be seen that the actual position starts to deviate from the expected position at t=7.6s. It can be considered that the controller can comply with the interaction force in 0.1s. Sample the first desired position at t=7.88s, which is the position that needs to be returned to after the first deviation. The actual position returned to −0.656 at t=13.09s, while the value of $K_{v}$ returned to 24.98 at t=9.92s. It can be concluded that it takes 3.17 (13.09–9.92) seconds to return to the expected position after the significant external force disappears. So, the strategy designed here can comply with large forces quickly and return slowly, ensuring both safety and comfort. Figure 21 shows the displacement of robot joint 2. The three deviation positions are 1.793, 1.471, and 1.401 rad. The same analysis process as for joint 1 can lead to the same conclusion.

## 4. Discussion

The customization trajectory combines the training experience of rehabilitation physicians with the different characteristics of patients. Therefore, the training trajectory is of physiological significance and more effective. Hou et al. designed a load-adaptive zero-force control algorithm based on joint torque sensors [29]. However, installing a sensor at every joint will make the structure complex. Additionally, the control algorithm is complicated. The robot system in this study is only equipped with a three-dimensional force sensor at the end to detect the HRI force. Therefore, the vertical load and the gravity of the third link can be detected and compensated directly. As for the first two links of the robot, there is no need to compensate for their gravity because they rotate in the horizontal plane. However, the static friction of the rotating joint is identified and compensated. Meanwhile, the interactive force is converted into the joint space by the Jacobi matrix and then the admittance algorithm is used to achieve easy dragging of the robot. The approach avoids the inverse kinematic model compared with the direct application of the admittance algorithm in Cartesian space. Most studies only filtered the original trajectory data to reduce jitters after obtaining the trajectory [30]. Although the high-frequency noise was removed, some extreme points corresponding to the range of joint motion of the upper limb may be deleted too. In this paper, the data compression algorithm can retain the outermost point of the original trajectory and maintain the topological shape of the trajectory, which will ensure the maximum motion position. Then the improved BOA algorithm is adopted to obtain an interpolation NURBS curve with the smallest sum of curvature. However, the velocity and acceleration planning of the interpolation curve are not carried out in this study and they are just generated by the derivative of the NURBS curve, which can be adjusted by changing the density of interpolating points and the time to complete the training trajectory. The maximum tracking error in trajectory tracking control is within 12mm, which may be due to the RBF network not being able to accurately approximate the dynamic model of the robot. The compliant control strategy can realize the compliant tracking property and slow returning movement. Compared with the parameter adaptation control strategy [31], the adaptability here may be inferior but the design of the controller is simpler, which is convenient for deployment and application.

## 5. Conclusions

In this paper, we introduced the self-developed upper limb rehabilitation training robot system briefly. The robot is a 3 DOFs end-effector robot, with the third prismatic joint vertical to the first two rotating joints. So, the motion of the third joint is uncoupled from the first two joints, which simplified the motion analysis and control. The trajectory customization with human–robot coupling is completed by load and friction compensation and admittance control. The customization forces are within 6 N making it easy to customize a personal training trajectory. The NURBS curve is used to interpolate between the compressed points and the curvature of the trajectory is optimized. It smooths the training trajectory and retains the topological shape of the original trajectory. Finally, the variable control gain algorithm based on the RBF network is designed. The proposed method ensures the trajectory tracking error within 12 mm and compliance with the large HRI force in 0.5 seconds to ensure the safety of passive training. With the method of error subdivision, the return movement after compliance is slow, making the patient feel comfortable. In the future, more useful rehabilitation modes will be studied and designed, especially the assisted-as-needed active rehabilitation strategy that is suitable for the patient who has regained some muscle strength.

## Figures and Tables

**Table 1:** Test functions.

Function	Dimension	Interval	Minimum
$f=x_{1}^{2}+x_{2}^{2}$	2	[−1010]	0
$f=x_{1}^{2}+2x_{2}^{2}-0.3\cos\left(3\pi x_{1}\right)-0.4\cos\left(4\pi x_{2}\right)+0.7$	2	[−1010]	0
$f=-20e^{-0.2\sqrt{(x_{1}^{2}+x_{2}^{2})/2}}-e^{(\cos\left(2\pi x_{1}\right)+\cos\left(2\pi x_{2}\right))/2}+20+e$	2	[−1010]	0
$f=|x_{1}\left|+\right|x_{2}\left|+\right|x_{1}\left|\right|x_{2}|$	2	[−1010]	0

**Table 2:** Maximum trajectory tracking error.

Direction	X	Y	Z
Maximum error	7.437mm	8.269mm	11.19mm

## Data Availability

All test data mentioned in this paper will be made available upon request to the corresponding author’s email with appropriate justification.
